# Potential Value of Wood Tar as a Natural Fungicide against *Valsa mali*

**DOI:** 10.3390/molecules27051531

**Published:** 2022-02-24

**Authors:** Yue Chen, Mengjing Lv, Juan Zhou, Ke Huang, Yubo Sun, Juntao Feng

**Affiliations:** Research and Development Center of Biorational Pesticides, Northwest A & F University, Yangling 712100, China; nwafuchenyue@163.com (Y.C.); lmj15382483676@163.com (M.L.); zhouj@nwafu.edu.cn (J.Z.); HK15991771058@163.com (K.H.); sybnongyaoxue@163.com (Y.S.)

**Keywords:** apple *Valsa* canker, botanical fungicide, field efficacy, wood tar

## Abstract

The *Valsa* canker caused by *Valsa mali* seriously harmed the production of East Asian apples and caused very significant economic losses. Considering the chemical residues and the improvement of people’s awareness of environmental protection, there is a need for screening new green pesticides for the control of *Valsa* canker. Therefore, we conducted systematic evaluations on the antifungal activity of wood tar. In this research, the effective concentration (EC_50_) of six strains of *V. mali* to wood tar was determined, and the EC_50_ ranged from 69.54 to 92.81 μg/mL. After treatment with wood tar, the hyphae of *V. mali* broke, swelled, and deformed; the permeability of the cell membrane increased; and the activity of pectinase reduced. Moreover, the expression levels of five genes related to pectinase also decreased significantly. In addition, the activities of phenylalanine ammonia lyase (PAL) and peroxidase (POD) of apple leaves treated with wood tar also increased. On detached apple branches, wood tar also showed therapeutic and protective activities. In the 2016–2019 field experiments, wood tar also showed good efficacy against *Valsa* canker and promoted the formation of callus. (In the experiments from 2016 to 2019, it can be seen that the control effect of 50% wood tar and 100% wood tar in the field is above 75% and promoted the formation of callus.) This study is the first to report the bidirectional efficacy of wood tar against *Valsa mali* and for trunk wound healing. The above results evidenced that wood tar has great potential to be developed as a natural alternative to commercial fungicides for the management of apple *Valsa* canker.

## 1. Introduction

Apple *Valsa* canker is a devastating disease caused by *V. mali*. In recent years, *V. mali* has seriously hindered the production of apples, with an average incidence of more than 50% in the Shaanxi Province of China, and the affected area is between 30% and 90%. *V. mali* infects branches, trunks, and fruit, causing necrosis of the diseased part, spreading to the entire trunk, and even causing the tree to the death in severe cases. It has caused great harm to apple production in East Asia, which is the main area of apple production, causing great economic losses to farmers. This pathogen can easily infect, especially when the tree is damaged or frostbite. Because the disease is occult and spreads rapidly, it is difficult to control the disease effectively [1].

In the past few decades, fungicides have been the main disease prevention and control agents. The asomate used on apple trees in the early years has been banned by the Ministry of Agriculture of China due to its high toxicity and high residue. According to recent reports, methionine acetic acid is recommended for the treatment of rot diseases, but it has not been registered [2]. Chemical agents have a limited ability to inhibit lesion expansion [3]. The risk of resistance also increases due to the high and excessive use of chemical fungicides [4]. When the fungicide is used for a long time, target pathogens easily develop resistance, and the iteration of the fungicide can not achieve the desired effect, so it is necessary to look for new alternatives in controlling the *Valsa* canker.

Wood tar is the byproduct that is discharged when wood is carbonized in a charcoal kiln or distilled in a distillation furnace. The application of wood tar is mostly limited to the scope of deep processing and mainly in the chemical industry, such as refining plasticizers and compounds used in chemical plants, which causes most of the content to be wasted. Although the antifungal activity of wood tar has been reported [5], its activity against *V. mali* is little known. The stickiness of wood tar is a fairly individual characteristic that can adhere to the lesion for a long time. Phenols are the main components of wood tar. Most of the phenols contained in it have bactericidal properties, such as phenol, 2-methoxyphenol, 2-methoxy-4-methylphenol, 2,6-dimethoxyphenol, and other reported antibacterial substances [6]. The synergistic action of various antibacterial compounds contained in wood tar can effectively slow down the occurrence of resistance. In addition, wood tar was natural and environmentally friendly.

Therefore, the purpose of this study is to: (a) evaluate the susceptibility of different samples of wood tar to *V. mali*, (b) explore the effect of wood tar on the morphological and physiological characteristics of *V. mali*, (c) estimate the protective and therapeutic activities of several tars on apple tree branches, and (d) assess the field efficacy of wood tar in controlling *Valsa* canker. These experiments will systematically evaluate the potential value of wood tar as a natural fungicide against *V. mali* and provide new information on understanding the mechanism of wood tar against *V. mali*.

## 2. Results

### 2.1. Sensitivity to Wood Tar

The EC_50_ values of wood tar inhibiting the mycelia growth of the six *V. mali* strains ranged from 69.54~92.81 μg/mL, with an average EC_50_ value of 81.16 ± 3.24 μg/mL. The EC_50_ value of carbendazim inhibiting mycelial growth ranged from 0.08 to 0.21 μg/mL, with an average EC_50_ value of 0.152 ± 0.019 μg/mL (Table 1).

### 2.2. Effect of Wood Tar on Mycelial Morphology

**Figure 1:**
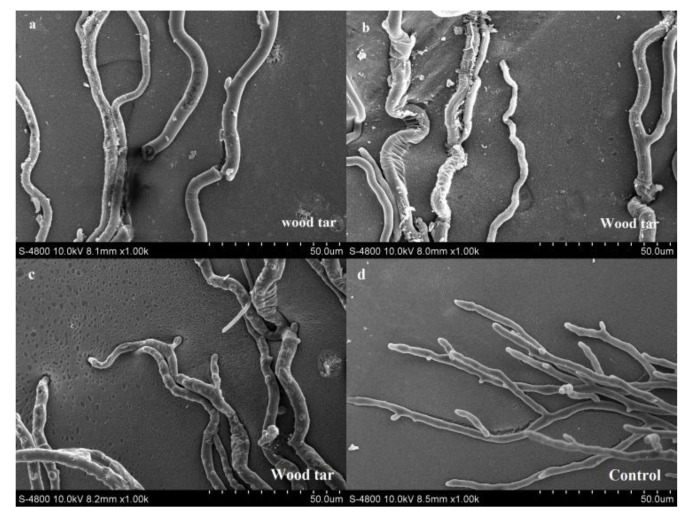
Effect of wood tar on the mycelial morphology of *V. mali*: (**a**–**c**) plates treated with EC_50_ concentration; (**d**): untreated plates.

A scanning electron microscope was used to observe the mycelial morphology of *V. mali* treated or untreated with wood tar. After treatment with wood tar with a concentration of EC_50_, the mycelia were broken, swollen, wrinkled and twisted (Figure 1a–c). The mycelia in the control group were smooth and grew normally (Figure 1d).

### 2.3. Effect of Wood Tar on the Permeability of Cells

**Figure 2:**
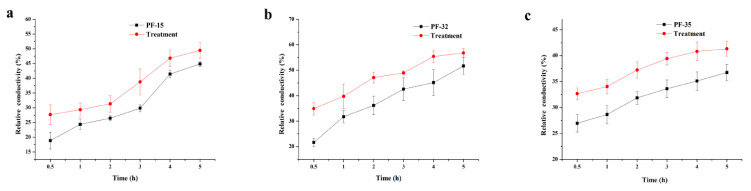
Effect of wood tar on the relative conductivity of the three strains (**a**) PF-15, (**b**) PF-32, and (**c**) PF-35. Bars denote the stand errors of three experiments.

Whether with wood tar treatment or not, the relative conductivity of the strains increased over time. After wood tar treatment, the relative conductivity of the three strains was always higher than the corresponding untreated control (Figure 2).

### 2.4. Effect of Wood Tar on Pectinase Activity and Oxalic Acid Content

**Figure 3:**
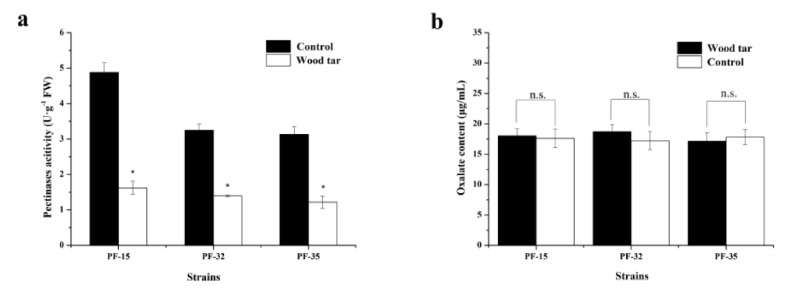
Effect of wood tar on the pectinase activity (**a**) and oxalic acid content (**b**). Bars denote the stand errors of three experiments. * denotes significant difference according to Fisher’s protected LSD test (*p* = 0.05).

The activity of pectinase in the three strains differed from each other. After treatment with wood tar, the activity of pectinase significantly declined compared to the control (Figure 3a). However, there was no significant difference in the content of oxalic acid when treated with wood tar or not (Figure 3b).

### 2.5. The Expression of Pectinase Genes

**Figure 4:**
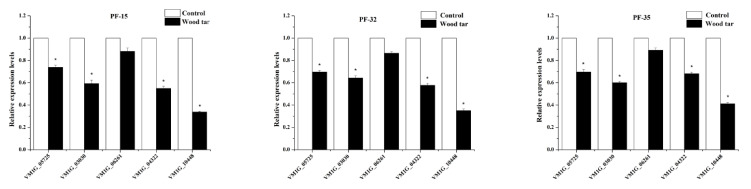
Relative expression levels of these five genes in three wild-type *V. mali* strains: PF-15, PF-32, and PF-35. The mean and standard error of three independent experiments is presented numerically. Asterisks indicate significant differences from controls according to Student’s *t*-test: * *p* < 0.05.

In this study, we examined the expression levels of five pectinase genes by q-RT-PCR [7]. As shown in Figure 4, four pectinase genes (*VM1 G_05725*, *VM1 G_03030*, *VM1 G_04322*, *VM1 G_10448*) were significantly down-regulated after wood tar treatment, while the gene (VM1 G_06261) had a slightly lower, but not significant, expression level after wood tar treatment (Figure 4).

### 2.6. Activity of PAL and POD in Apple Leaves

**Figure 5:**
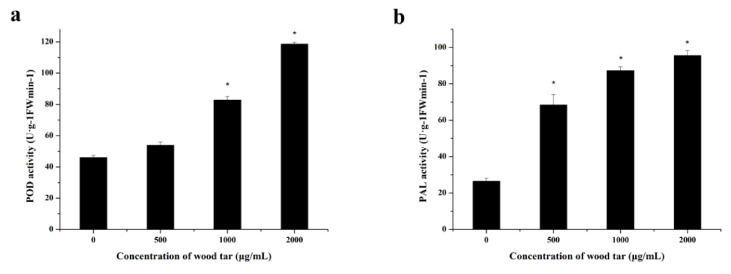
Effect of wood tar on the POD (**a**) and PAL (**b**) activity in apple leaves. Bars denote the stand errors of three experiments. Asterisk denotes significantly different from the control according to Student’s *t*-test: * *p* < 0.05.

As shown in Figure 5, the POD and PAL activities of apple leaves gradually enhanced with the increased concentration of wood tar.

### 2.7. Protective and Therapeutic Activity of Wood Tar on V. mali

**Figure 6:**
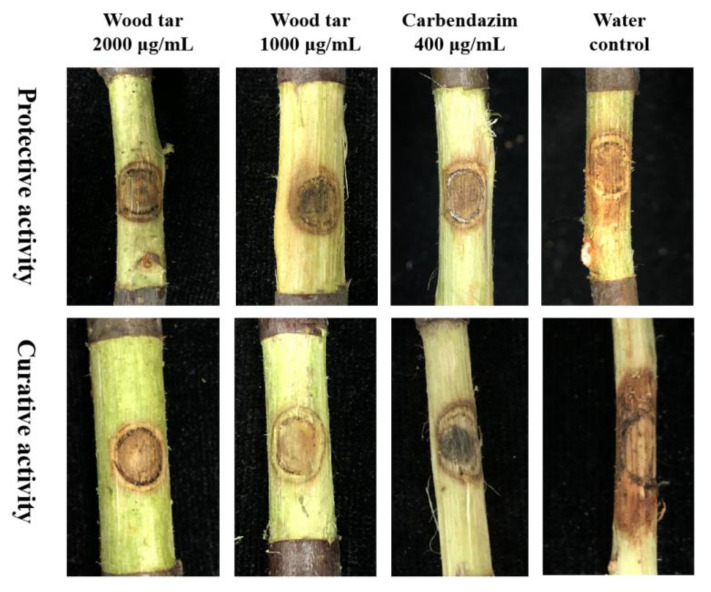
Protective and curative activity of wood tar against *Valsa* canker.

After inoculation for 3 d on detached apple branches, a larger area of lesions was produced by the strain PF-32 and treated with water. When treated with 2000 μg/mL wood tar before inoculation, the protective efficacy reached 77.35%, which was comparable to that of carbendazim at 400 μg/mL (Table 2). When treated with 2000 μg/mL wood tar after inoculation, the curative effect was 67.89%. In addition, 1000 μg/mL wood tar had more than 45% protective and therapeutic activity against *Valsa* canker. These results showed that wood tar has both protective and therapeutic activity (Figure 6).

### 2.8. Field Test of Wood Tar on Valsa Canker

**Figure 7:**
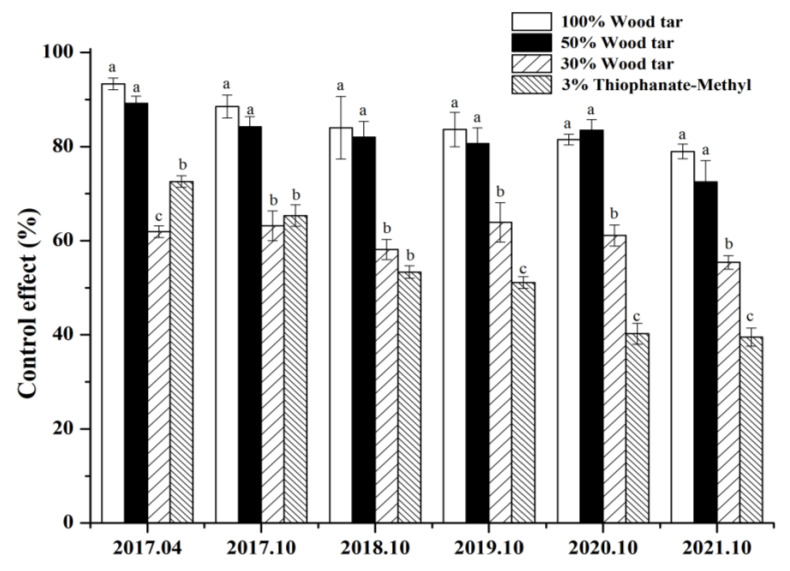
Field efficacy of wood tar against *Valsa* canker. Bars denote the stand errors. Different letters above the columns indicate significant differences according to Duncan’s multiple range test (*p* < 0.05).

**Figure 8:**
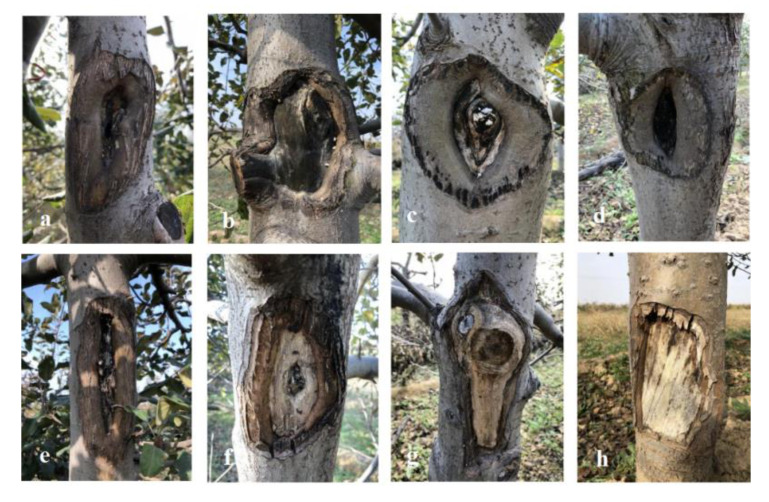
Field efficacy of wood tar on *Valsa* canker: (**a**–**f**) pictures of the diseased parts of the trunk in 2021; (**g**,**h**) field pictures of control in 2021.

After being treated with 50% concentration, 30% wood tar, and 3% Thiophanate-methyl, the 50% concentration of wood tar is significantly more effective than 30% wood tar and 3% Thiophanate-methyl in preventing *Valsa* canker in four years (Figure 7). From Figure 8, it can be seen that the control results of wood tar on the diseased part of *Valsa* canker are more significant compared with the control, and the effect of promoting healing is also very obvious.

## 3. Discussion

Apple *Valsa* canker is still a serious disease threatening the main apple-producing areas. The commonly used fungicides have not been able to achieve the ideal control effect, and the problems of residue and drug resistance became increasingly prominent [8,9]. The selection of substances that are environmentally friendly, stable in physical properties, and have bactericidal activity has become a point of attention [5,10,11,12]. Wood tar has a long history of use, and it was used for wood preservation in ancient times due to its ability to penetrate the xylem [5].

In this study, the sensitivity of wood tar to six different strains of *V. mali* was determined. The results suggested that wood tar exhibited sufficient antifungal activity against *V. mali*. In this study, wood tar treatment increased the cell membrane permeability, indicating that wood tar may disrupt the cell membrane structure of *V. mali*, leading to cell membrane rupture and leakage. Previous studies have shown that the virulence and the content of oxalic acid are closely related. Oxalic acid is necessary for pathogenicity [13]. In addition, pectinase plays a key role in the infection of apple bark and is one of the cell wall hydrolases secreted by *V. mali* [7]. In this study, the level of pectinase activity decreased after wood tar treatment, indicating that wood tar inhibited the ability of *V. mali* to infect apple trees. In this study, wood tar had both good protective and therapeutic activity against *V. mali*. Moreover, the protective activity was always better than that of curative activity, which indicated that wood tar should be used as a protective agent to better prevent the occurrence of the disease.

There is evidence that POD and PAL are key enzymes involved in plant defense [14,15]. In the presence of hydrogen peroxide, POD can oxidize phenol to quinine. PAL participates in the synthesis and accumulation of plant secondary disease resistance substances (phytoalexin, lignin, and phenolic substances) [16,17]. The formation of lignin can increase the thickness of cell walls and the degree of tissue lignification and form a mechanical barrier for pathogen invasion. After wood tar treatment, the activities of POD and PAL increased. This is consistent with the results in Figure 6 that the callus after treating the lesion with wood tar is thicker than the untreated tissue. It is of important research significance to study whether wood tar has the function of plant resistance induction. Due to winter pruning and scraping of diseased spots, *V. mali* invaded. The application of wood tar can not only protect the wound but also inhibit the expansion of *V. mali*.

The results of the field tests from 2017 to 2020 show that wood tar has a significant effect in controlling the annual infection of *V. mali*, and the control effect in four years has obtained unexpected results in the field. The results show that 100% and 50% wood tar demonstrated a better controlling effect, and there was no significant difference between their efficacy. Importantly, different types of wood tar showed lasting efficacy. In the first two years after application, the efficacy of the reference fungicide was slightly higher than the treatment group, while in the latter three years, the effect was reversed. The results of field experiments are affected by multiple factors. In the future, we will continue to conduct more field trials to further clarify the use method and dose of the wood tar.

Pectinase is one of the important cell wall hydrolase enzymes in *V. mali* infecting process. Pectinase invades and colonizes apple tissue by hydrolyzing apple cell walls. According to previous studies, after knocking out pectinase-related genes, the pathogenicity of *V. mali* was significantly reduced [18]. In this study, the activity of pectinase was significantly decreased after treatment with wood tar, and the levels of five genes related to pectinase were also down-regulated to varying degrees. The above results prove that wood tar can reduce the virulence of *V. mali* infections in apples by inhibiting the activity of pectinase and the expression level of related genes.

It is considered one of the environmentally friendly methods to find new ways to use from discarded plant resources. Because of its stable and abundant carbon source, wood tar is also a new type of pesticide that can provide nutrients to plants. Although wood tar has a complex composition, it has research prospects due to its excellent antibacterial activity and induced resistance.

In summary, this study confirmed that wood tar has antifungal activity, can inhibit the onset of plants for a long time, has a good healing effect on wounds, and can be developed as a new plant fungicide. As far as we know, this is the first report of the antifungal activity and biochemical reaction of wood tar to *V. mali*, which will provide new research ideas for the mechanism of wood tar. The mechanism of wood tar against *V. mali* was complex. It can be seen from the results that wood tar inhibits the activity of the pathogen by affecting the activity of pectinase, a key enzyme in *V. mali* infecting apples. This is also corroborated by the down-regulation of pectinase-related genes in *V. mali*. Further studies are still needed to explore the mechanism of wood tar against *V. mali*.

## 4. Materials and Methods

### 4.1. Fungicides, Medium, and Strains

Wood tar (100%) was purchased from Shaanxi Yixin Bioenergy Technology Development Co. Ltd. and dissolved in 10 mL ethanol to 100 mg/mL. Carbendazim (98%) provided by Shenyang Institute of Chemical Industry (Shenyang, China) was dissolved in 0.1 mol/L hydrochloric acid (hydrochloric acid).

Potato dextrose agar (PDA) was prepared with 200 g of potato, 18 g of agar, and 20 g of glucose per liter of distilled water. Six pure monospore strains collected from Shaanxi Province, China, were provided by the College of Plant Protection of Northwest A&F University and kept at 4 °C on PDA. The six *V. mali* strains in this study were collected from different apple branches in Shaanxi Province. The surface of apple branches with *Valsa* canker symptoms was cut and sterilized with 1% NaClO for 1 min, then treated with sterile water for 30 s and air-dried. The sterilized apple branches were placed on PDA plates and soaked 3 times with 100 μg/mL NaClO at 25 °C. These pure cultured *V. mali* strains were isolated and identified based on previous studies [19]. All these strains were maintained on the PDA slide at 4 °C prior to sensitivity testing.

### 4.2. Sensitivity of V. mali to Wood Tar

The sensitivity of *V. mali* to wood tar was tested as follows. Mycelia plugs (diameter 4 mm) taken from the colony of *V. mali* were placed on PDA plates containing 0, 6.25, 12.5, 25, 50, 100, and 200 μg/mL of wood tar. The plates were placed in an incubator at 25 °C. After 5 days of inoculation, the diameter of the colony was measured, and the EC_50_ value was calculated [20]. There are three PDA plates for each treatment. In this test, carbendazim was used as the reference fungicide, and the concentration was as follows: 0, 0.0625, 0.125, 0.25, 0.5, 1, and 2 μg/mL.

### 4.3. Effect of Wood Tar on Mycelial Morphology

The mycelia plugs extracted from the edge of the vigorously growing colony of the PF-15 strain were transferred to the PDA plate containing the EC_50_ value of wood tar. Take PDA without wood tar as a control. After 4 days, in a 25 °C incubator, the edge of the area (10 mm x 10 mm) was cut, specimens were fixed in 4% glutaraldehyde diluted in 0.1 mol/L phosphate buffer (pH 6.8) for 6–8 h at 4 °C, washed with the same buffer for 1 h, dehydrated with graded ethanol concentrations (30, 50, 70, 80, 90, 90, and 100%) for 20 min, critical point dried, mounted on stubs, and sputter-coated with a platinum-plated palladium target sand [19,21]. The hypha morphology of *V. mali* was observed with a scanning electron microscope (SEM, JSM-6360 LV, Japan) [22].

### 4.4. The Effect of Tar on the Cell Membrane Permeability

For the three strains PF-15, PF-32, and PF-35, 10 mycelia plugs were transferred to a 250 mL bottle containing 100 mL of PDB. After shaking at 180 rpm for 72 h, the EC_50_ value of some flasks was corrected with cuminic acid at 25 °C for 72 h. A flask without cumin acid was used as a control. After shaking the flask for 24 h, the hyphae were collected. Then, 0.5 g of fresh mycelium per sample was suspended in 25 mL of distilled water. We measured the conductivity of distilled water after 0.5, 1, 2, 3, 4, and 5 h with a conductivity meter (CON510 Eutech/Oakton, Singapore). After 5 h, the mycelium was boiled for 5 min, and the final conductivity was measured. There are three replicates for each treatment, and the experiment is three times in total. We used the following equation to calculate the relative conductivity of *V. mali* [23]:

Relative conductivity at different times = conductivity × 100%

### 4.5. Pectinases Activity and Oxalic Acid Content

Pectinases activity was determined according to Wang et al. [23]. Briefly, different volumes of D-galacturonic acid solution (0, 0.2, 0.4, 0.6, 0.8, or 1.0 mL) added with double steam water to 1 mL. Then, we added 3.0 mL of 3, 5-dinitrosalicylic acid and 0.5 mL of citric acid buffer (pH 4.8) and boiled it for 5 min. After natural cooling, a constant volume was reached by double steaming water to 5 mL. The absorbance at 540 nm was measured with a microplate reader, and the standard curve was drawn.

In order to determine the pectinase activity, the above filtrate was centrifuged at 4 °C at 12,000 rpm for 5 min. Then, the activity of pectinase in the supernatant was determined according to the standard curve. Oxalic acid content in the supernatant above was determined according to [24]. Three replicates per treatment were used, and the experiment was conducted three times.

### 4.6. Effect of Wood Tar on the Expression of Genes Involved in Pectinase

Total RNA was extracted with a kit (Omega Biotechnology, New York, NY, USA). Real-time quantitative polymerase chain reaction (qRT-PCR) was performed on a CFX96 Touch Real-Time PCR Detection System (Bio-Rad, Richmond, CA, USA). The expression of the pectinase gene was normalized to the reference gene G6 PDH [18]. Calculations were performed using the 2-ΔΔCt method [25]. qRT-PCR primers are in Table 3. Each treatment was repeated 3 times, and the experiment was performed 3 times.

### 4.7. Effect of Wood Tar on the Activity of POD and PAL

Apple leaves cut from a three-year-old Fuji tree were sprayed with 1000 and 2000 mg/L of wood tar until there was runoff. After air drying, the leaves were ground in liquid nitrogen, and the activity of POD and PAL was determined using a commercial kit according to previous studies [26,27]. Ten leaves were used for each treatment, and the experiment was repeated three times.

### 4.8. Protective and Therapeutic Activity of Wood Tar against V. mali

The length of the detached branches from apple trees (10-year-old “Fuji trees”) was 10 cm. For protective activity, the branches were sprayed with water (control), wood tar at 1000 and 2000 μg/mL, and carbendazim at 400 μg/mL until runoff. After 24 h, mycelia plugs taken from the margin of the colony of *V. mali* strain 06−5 (selected at random) were placed on the branches and maintained at 25 °C. For therapeutic activity, fungicides were sprayed after 24 h of inoculation. After the branches were placed in a plastic box at 25 °C and 80% relative humidity for 72 h, the colony diameters in two vertical directions were measured, and the lesion area was calculated [28,29,30,31]. There are three plants with nine twigs for each treatment (three twigs are cut from one plant), and the experiment was repeated twice.

Lesion area (cm^2^) = long lesion × short lesion length × ¼

### 4.9. Field Test of Wood Tar against V. mali

This experiment was conducted in Dali County, Weinan City, Shaanxi Province. A total of five treatments were designed, including wood tar (30%, 50%, and 100%), 3% thiophanate-methyl ointment, and a blank control (treated with water). Fifty disease lesions of similar sizes were selected for each treatment. First, we used a scalpel to extend a 0.5 cm circle around the diseased spot and then a knife to thoroughly clean the diseased tissue and healthy skin inside the circle (the knife must be disinfected) to reach the xylem. We applied the drug evenly with a toothbrush around the spot of the diseased tissue (thickness about 2 mm) [32,33,34,35,36]. The experiment was conducted in the autumn of 2016, and the results were first investigated in the spring of the following year. After that, the results were investigated in October each year from 2017 to 2021.

### 4.10. Data Analysis

All the data were subjected to an analysis of variance (ANOVA) using SPSS 19.0 (SPSS Inc., Chicago, IL, USA). Duncan’s multiple range test was used to analyze the significant differences. The EC_50_ values were calculated using the Data Processing System (DPS, version 7.05) by plotting the percentage of mycelia growth inhibition against the log_10_ fungicide concentrations in three independent measurements.

## Figures and Tables

**Table 1:** The sensitivity of *V. mali* to wood tar and carbendazim.

Strains	EC_50_ (μg/mL) for	
	Wood Tar	Carbendazim
PF-15	76.73 bc ^a^	0.15 b
PF-18	83.45 b	0.13 bc
PF-24	85.20 b	0.19 c
PF-28	92.81 a	0.21 c
PF-32	69.54 c	0.08 a
PF-35	79.22 b	0.15 b

^a^ Mean values followed by the same letter within the same column were not significantly different in LSD (least significant difference) tests at *p* = 0.05.

**Table 2:** Protective and curative activity of wood tar in controlling *V. mali*.

Sample	Protective Activity		Curative Activity	
	Lesion Area (cm^2^)	Control Efficacy (%)	Lesion Area (cm^2^)	Control Efficacy ^b^ (%)
Wood Tar (2000 μg/mL)	1.17 a ^a^	77.35 a	1.813 a	67.89 b
Wood Tar (1000 μg/mL)	2.055 b	60.22 b	2.968 b	47.43 c
Carbendazime (400 μg/mL)	1.33 a	74.25 a	1.426 a	74.74 a
Control	5.166 c	-	5.646 c	-

^a^ Values followed by the same letter within the same column were not different according to Fisher’s least significant difference (LSD) (*p* = 0.05). ^b^ Control efficacy = [(Lesion area of control -Lesion area of treated group)/(Lesion area of control)] × 100%.

**Table 3:** qRT-PCR primers used in this study.

Primer	Sequence (5′–3′)	Use
P1	CTCGCCCATGTACTATGTCTTC	The qRT-PCR primer was
P2	GTATCCCAGCCATCCGTATTC	for the *VM1 G_05725* gene
P3	CCCGCACTACTTCTTCTTTGA	The qRT-PCR primer was
P4	ACGTCGCTTCCTTGGATTT	for the *VM1 G_03030* gene
P5	GTGACCATCTCTAACAGCCATATC	The qRT-PCR primer was
P6	CTGGTCATCAGATCCCGTAAAG	for the *VM1 G_06261* gene
P7	GGAGGGAATAAGGTGAGGATCTA	The qRT-PCR primer was
P8	CAGGCCTCAAGTACGCATTAT	for the *VM1 G_04322* gene
P9	CCAAAGTTCTTCAAGGCCAATC	The qRT-PCR primer was
P10	GCAGGTTGTTGATGTAGTTGATG	for the *VM1 G_10448* gene
P11	TCAGAACAAGTTCGAGGGCGACAA	The qRT-PCR primer was
P12	TGAGGGCAATAGAGGGCTTGTTCA	for the reference gene (G6 PDH gene)

## Data Availability

Not applicable.
